# Complementing the Eukaryotic Protein Interactome

**DOI:** 10.1371/journal.pone.0066635

**Published:** 2013-06-18

**Authors:** Robert Pesch, Ralf Zimmer

**Affiliations:** Institute for Informatics, Ludwig-Maximilians-Universität München, Munich, Germany; Institute for Research in Biomedicine, Spain

## Abstract

Protein interaction networks are important for the understanding of regulatory mechanisms, for the explanation of experimental data and for the prediction of protein functions. Unfortunately, most interaction data is available only for model organisms. As a possible remedy, the transfer of interactions to organisms of interest is common practice, but it is not clear when interactions can be transferred from one organism to another and, thus, the confidence in the derived interactions is low. Here, we propose to use a rich set of features to train Random Forests in order to score transferred interactions. We evaluated the transfer from a range of eukaryotic organisms to *S. cerevisiae* using orthologs. Directly transferred interactions to *S. cerevisiae* are on average only 24% consistent with the current *S. cerevisiae* interaction network. By using commonly applied filter approaches the transfer precision can be improved, but at the cost of a large decrease in the number of transferred interactions. Our Random Forest approach uses various features derived from both the target and the source network as well as the ortholog annotations to assign confidence values to transferred interactions. Thereby, we could increase the average transfer consistency to 85%, while still transferring almost 70% of all correctly transferable interactions. We tested our approach for the transfer of interactions to other species and showed that our approach outperforms competing methods for the transfer of interactions to species where no experimental knowledge is available. Finally, we applied our predictor to score transferred interactions to 83 targets species and we were able to extend the available interactome of *B. taurus*, *M. musculus* and *G. gallus* with over 40,000 interactions each. Our transferred interaction networks are publicly available via our web interface, which allows to inspect and download transferred interaction sets of different sizes, for various species, and at specified expected precision levels. **Availability:** http://services.bio.ifi.lmu.de/coin-db/.

## Introduction

Using high-throughput screening techniques such as Yeast Two Hybrid screens, mass spectrometry and protein microarrays large amounts of protein interaction data can be obtained. A protein interaction consists of proteins which bind permanent or transient together in order to carry biological functions. Interaction data collected in various databases has for example been used to study regulatory networks, to explain experimental data or to predict the functions of proteins [1]. Researchers can query protein interactions in databases like IntAct[2] or BioGrid[3], which include interactions derived from large-scale experiments, from literature curations, from user submissions, and interactions from protein structures. Besides this, repositories exist which integrate the interactions from multiple sources. The current protein interaction networks are derived from high-throughput experiments and hypothesis-driven low-throughput experiments applied to particular gene sets of interest[4].

But still the experimental identification of interactions is a time consuming and costly process, so that high-throughput experiments have mostly been conducted on model organisms such as *S. cerevisiae* [5], *H. sapiens* [6], *A. thaliana* [7] and *D. melanogaster* [8]. Thus, the available interaction networks for other species are extremely sparse (see Table 1).

**Table 1:** Protein interaction networks.

Species		Interactions				
	Genes	Physical	Genetic	Other	Total	Total interactions
						per gene
S. cerevisiae	6,328	55,767	104,926	17,674	178,367	28.19
H. sapiens	28,383	43,412	71	20,992	64,475	2.27
D. melanogaster	14,321	19,088	2,118	17,265	38,471	2.69
S. pombe	4,958	1,943	9,665	804	12,412	2.5
C. elegans	20,184	5,483	1,785	4,208	11,476	0.57
M. musculus	24,865	3,513	3	2,596	6,112	0.25
A. thaliana	26,496	5,048	67	937	6,052	0.23
P. falciparum	5,503	2,215	0	4	2,219	0.4
R. norvegicus	24,770	804	0	867	1,671	0.07
D. rerio	24,352	173	11	13	197	0.01

Overview of protein interaction networks extracted from the database iRefIndex [33] for the ten eukaryotic model species with the largest protein interaction networks. Besides the total number of protein interactions, the number of physical, genetic and interactions with an unknown interaction type is given. Only the interaction network of *S. cerevisiae*, *H. sapiens*, *D. melanogaster* and *S. pombe* have more than 2 interaction per gene (*S. cerevisiae* peaks with 28.19).

Furthermore, all experimental protein interaction detection methods have different weaknesses and biases[9]. For example false positive rates up to 50% are reported for Yeast Two Hybrid screens [10], literature curations do only agree to some extent [11], and interactions from Tandem Affinity Purification (TAP), which requires further processing in order to infer physical protein interactions[12], [13].

Numerous computational approaches have been developed to predict protein interactions. In particular, knowledge from other (model) organisms can be used to predict protein interactions for a specific target organism. But link attachments, link detachments, gene duplications and gene losses lead to (evolutionary) changes in protein interaction structures [14]. For example, single nucleotide substitutions in the coding region of a gene can lead to structure changes of the encoded protein so that new binding partners can dock or other proteins can not dock anymore to the particular protein. Gene duplications lead also to duplications of interactions and again nucleotide substitutions of the genes lead to a network rewiring. While transient protein interactions are affected by rewiring, gene gains and losses, protein complexes are mostly affected by losses and gains of subunits [15], [16].

Matthews et al. [17] introduced the term interolog (an orthologous gene pair interacting in at least one species) and many methods transfer interaction data employing interologs [18]–[22]. Matthews et al. was able to experimentally validate between 16% to 32% of transferred protein interactions from *S. cerevisiae* to *C. elegans* with different ortholog identification techniques. Several features are commonly used to increase the reliability of interaction transfers via interologs. The simplest approach is to require a certain interolog quality, e.g. a minimum bootstrap score for orthologs from the InParanoid database [18] or a minimum sequence similarity between orthologs in order to transfer an interaction. Yu et al. showed that protein interactions can be safely transferred if the joint sequence identity between the orthologs involved in the transfer is larger than 80% [22]. More advanced filter approaches use thresholds for the Gene Ontology (GO) [23] annotation similarity, domain similarity, gene expression correlation or other features of the interologs [20], [21], [24]–[26]. To achieve a specified performance, random protein pairs are compared with known protein interaction partners to define thresholds for the different features. Besides the inference of protein interaction from interologs, various other approaches try to predict interactions using structural properties [27], network topology information [28], or protein domain information [29]. The *STRING* database follows a different approach to score interactions by combining information from experiments, databases, text-mining and transfer information [30].

Lewis et al. showed that the transfer consistency cannot easily be improved. Furthermore, they showed that the evolutionary change of interactions is too high to allow the direct transfer of interactions for phylogenetically distant species unless a strict definition of homology is used [31]. In contrast van Dam et al. showed that protein complexes are highly conserved even between *H. sapiens* and *S. cerevisiae* [15]. All network transfer studies relay on homologies which can be identified with different ortholog detection methods like simple bidirectional BLAST best hit results, graph-based methods that cluster orthologs, or tree-based methods. Benchmarks of orthologs detection methods have shown that there is no best method for ortholog detection [32]. It is obvious that with conservative ortholog detection approaches only relatively few interactions can be transferred, but that these interactions are more likely conserved, whereas with cluster based and tree based methods groups of orthologs are produced which allow to transfer more interactions. Thus, the usage of ortholog identification approaches, the choice of experimental data (only physical interaction derived from Yeast Two Hybrid studies, or more relaxed interaction data which includes interactions from TAP or Co-Immunoprecipitation experiments, or even protein complexes), and the approaches used to deal with the incompleteness of current networks result in different estimated protein interaction conservation rates.

In this paper, new features and successfully used features in the literature are exploited to train Random-Forests-Filters (*RFF*) for the reliable transfer of interactions to even distant species. The *RFF* models are trained with interactions transferred from various eukaryotic species to *S. cerevisiae* using all available interactions from an integrated database and orthologs from cluster based approaches. We train the models on yeast for the only reason that the *S. cerevisiae* network is assumed to be the most complete one, which allows to distinguish correct and incorrect transfers in the learning phase. Another assumption we make is that the learned *RFF* models can be used for other species as well. This is reasonable as the models learn the important features (e.g. sequence similarity, orthology, network properties, functional similarities) and their appropriate weightings, which will hold in a species-independent way (there are no particular *S. cerevisiae* specific features or parameters). The transfer performance on *S. cerevisiae* is taken as an estimate for the expected performance on other species, especially for phylogenetically closer ones. We applied the trained *RFF* predictor to transfer interactions on a large scale in-between various eukaryotic species. This increases the available reliable interactions for non-model organisms manyfold without inflicting too many false positives. The transferred networks are publicly available at our web interface. Compared to competing approaches to predict protein interactions we integrate a wide range of features and, instead of using fixed thresholds, employ a systematic and conservative *RFF* approach with an associated performance estimate for the (distant) transfer to *S. cerevisiae*.

## Materials and Methods

### Data sources

We use an integrated database to get a complete view on the currently discovered protein interaction networks and use the interaction repository iRefIndex[33] as the source database for experimentally determined protein interactions. iRefIndex provides interaction data for multiple species in a common format from the 13 different interaction databases: BIND[34], BioGRID[3], CORUM[35], DIP[36], HPRD[37], InnateDB[38], IntAct[2], MatrixDB[39], MINT[40], MPact[41], MPIDB[42], MPPI[43] and OPHID[44]. All databases include experimental validated data extracted from different sources, besides OPHID which also makes use of transferred interactions. Therefore, we excluded interactions from OPHID for our study. Furthermore, iRefIndex includes binary interactions (physical and genetic) and few protein complexes. We transfer binary interactions from iRefIndex (physical, genetic and other interaction types including ambiguous or interactions without type annotation) to target species using publicly available ortholog mappings. Orthologs are obtained from the Orthologs Matrix Project (OMA) [45], InParanoid [46] and HomoloGene [47]. These databases are used due to the evaluation results in [32] and the coverage of ortholog mappings for various eukaryotic species. The interaction partners and orthologs are mapped to UniProt [48] as a common reference to obtain annotations including GO terms, synonyms and mappings to external databases (see Table S1 for an overview of the used data sources). We consider all eukaryotes species for which we could transfer at least one interaction given the interaction and ortholog databases. Thus, we consider 83 out of the approximate 166 (until January 2013) fully sequenced eukaryotes for the subsequent analysis (for a list of sequenced eukaryotes see EBI Genomes Pages: http://www.ebi.ac.uk/genomes/eukaryota.html).

### Interaction transfer

Protein interaction networks are modeled as graphs 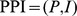 consisting of a set of proteins 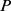 and interactions 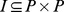. Given an interaction network 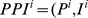), a target protein set 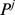 and an ortholog mapping 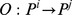, a *(directly) (interolog based) transferred interaction network* consists of 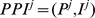 with 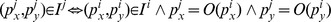. Transferred interactions 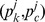 can be scored and filtered to obtain a *(filtered) (interolog based) transferred interaction network*. In our case, a trained Random Forest Filter (*RFF*) model is used and its performance for specific score thresholds is estimated via the transfer data to *S. cerevisiae*.

### Random-Forest-Filter (*RFF*)

For the scoring of transferred interactions we use Random Forests (RF) from the WEKA [49] machine learning framework. Random Forests predict the outcome class (correct, incorrect) of an instance (transferred interaction) by using a voting procedure on several learned decision trees with different feature sets. Random Forests have shown good evaluation results on similar learning tasks [50] and are considered more robust against noise than other ensemble machine learning methods [51]. RF rely on two parameters, the number of trees to learn and the number of features to consider. We determine these parameters via a grid search. In addition to the output class label, the WEKA Random Forest implementation provides a score value between 0 (low confidence) and 1 (high confidence), which we use as score value for transferred interactions.

### Features

As features we use the protein annotations of the interacting partners in the source and the target network and of the orthologs from which an interaction is transferred. The features can be classified into four categories: 1.) Features modeling Gene Ontology similarities (Gene Ontology), 2.) features derived from the network structure (Network), 3.) features describing the similarity between orthologs (Orthologs) and 4.) general features (General).

### Gene Ontology

#### GO similarity

We compute the semantic GO similarity for two proteins based on Resnik[52] information content measure
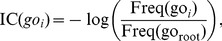
(1)with 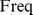 as the number of proteins annotated with a given term 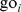, or its descendant terms in the GO tree. For two GO terms 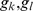, we define the semantic GO term similarity as the 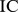 for their common ancestor. And for two proteins 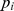, 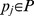 we define the semantic GO similarity as the maximum of all combination of GO annotations for the two proteins. Formally defined as
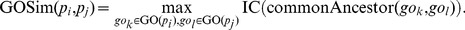
(2)


Given that measure, the semantic similarity is computed for the interaction partners in the source and target network and the orthologs. Besides a global semantic GO similarity, one feature is modeled for each of the GO categories cellular component, biological process and molecular function (indicated with C, B, and M behind the feature name in the following) to take the different types individually into account.

### Network

#### Network overlap

The overlap of the neighborhood proteins for a given pair of proteins in the source and target network. For this purpose the Jaccard Index is computed for the direct neighbors of the interacting proteins with the equation
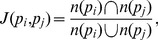
(3)where 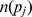 and 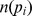 are the adjacent proteins in the protein interaction network.

#### Network GO similarity

The average semantic GO similarity between the pair-wise neighbors of the interaction partners in the networks computed with the equation
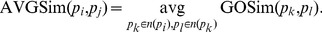
(4)


### General

#### Source interaction database

The source database from which an interaction is extracted as provided as additional information in the used integrated protein interaction database.

#### Edge support

The number of PubMed abstracts given as evidence for the source interaction.

#### Source interaction type

The source interaction type (physical, genetic or other) is used as discrete feature value. For this purpose the molecular interaction type [53] is used.

#### Total support

The number of times an interaction is transferred from all other networks to the target network as suggested by [54] for confidence scoring.

#### Gene expression correlation coefficient

Given a gene expression time series for two genes the Pearson correlation coefficient is computed for the putative interacting partners in the target network with the equations
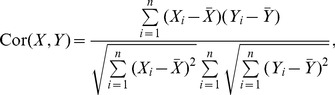
(5)where 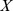 and 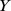 represent the expression values for the respective genes.

### Ortholog

#### Sequence similarity

The sequence identity of the orthologs.

#### Harmonic sequence similarity

The harmonic mean of the sequence identities of the orthologs.

#### Synonym similarity (Token score)

From the orthologs the function of the proteins is extracted from the textual description using UniProt[48] by tokenizing, stemming and filtering stop words and to general words resulting in a set of tokens which are descriptive for the proteins. Based on these function terms we define the similarity for two proteins 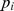 and 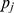 from the set of all protein 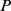 as
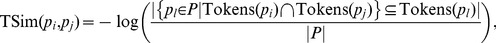
(6)where 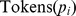 and 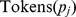 are the function terms of the proteins 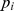 and 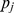.

#### Domain/Family similarity

The InterPro and PFAM annotations from UniProt[48] are used to compute the domain/family similarity of the orthologs. For two proteins we define the domain/family similarity as
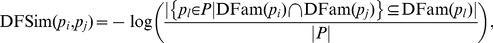
(7)where 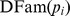 and 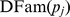 are the domain and family annotations.

#### KEGG Pathway score

Boolean indicator whether the orthologs are involved in the same pathways or in different pathways.

#### Ortholog source

The database from which the orthologs used for the transfer are extracted.

#### Ortholog score

Given two orthologs 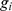 and 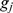 we define the ortholog score as
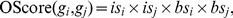
(8)where 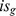 is the inparalog score and 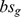 the bootstrap score provided by InParanoid for each gene 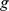 in a ortholog clusters.

#### Ortholog support

The number of times the same ortholog relation between two genes can be found in the different ortholog databases.

#### Phylogenetic distance

The distance of the source and the target species in a phylogenetic tree provided by [45].

#### Transitive ortholog

The idea behind this feature is that more conserved orthologs can be traced from a source species to a target species along the phylogenetic tree. For this purpose a phylogenetic tree covering all species with ortholog mappings is used. Given such a tree, a path from a source to a target species is computed by:

1. searching the shortest path between the two species and
2. searching the closest leaf nodes for all inner nodes on the shortest path.

The result is a list of species which are “between” the target and the source species. An ortholog is defined as transitively consistent if a direct ortholog between the source and the target species can also be reached when going along the pairwise ortholog mappings on the estimated path.

In the case that a feature cannot be computed because of missing annotation data, the feature is replaced by a missing value indicator. Features are derived from different sources. In the rest of the article we indicate with (T), (S) and (O) after the feature name whether a feature is modeled between the protein pair in the target network, the source network, or between the orthologs, respectively.

### Evaluation measures

To assess the quality of the learned models we compute the



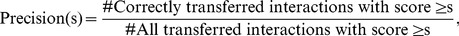
(9)




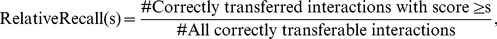
(10)and



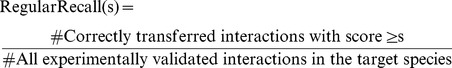
(11)for a given 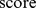 value assigned by the learned model. A precision of 1.0 for a given score threshold 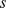 is obtained when all transferred interactions with a score value 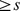 can be found in the experimentally validated network. The relative recall is 1.0 when all correctly transferable interactions using the available ortholog relations are also transferred after the filtering i.e. all transferred interactions have a score value 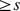. We mostly use the relative recall instead of the regular recall in order to assess the recall with respect to a direct transfer. As overall measure for different score thresholds the area under the precision (relative) recall curve (AUPRC) and the area under the receiver operating characteristic curve (AUROC) are used [55]. Furthermore, the Information Gain (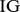) i.e. the reduction of entropy of the data set given information about a feature [56], is computed to estimate the impact of the different features.

Formally, for a data set 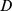 and feature 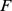 the IG is defined as
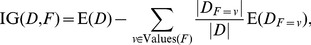
(12)where 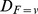 is the set of instances in 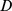 with value 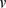 for feature 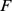 and 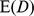 defined as
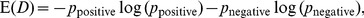
(13)where 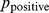 and 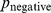 is the proportion of D belonging to the class of correctly (consistently) and incorrectly (inconsistently) transferred interactions, respectively.

## Results

### Current Protein Interaction Networks

Table 1 gives an overview of the protein interaction networks derived from the integrated interaction database iRefIndex having the largest number of interactions.

Over 78% of the *S. cerevisiae* and over 90% of the *D. melanogaster* interactions stem from high-throughput studies where over 1,000 interactions are reported, whereas for *H. sapiens* only 43% of the interactions stem from high-throughput studies (see Figure S1). Furthermore, most interactions for *S. cerevisiae* are detected with genetic interference and affinity chromatography technology methods like Co-Immunoprecipitation or Tandem Affinity Purification, whereas for *D. melanogaster* most interactions are detected within one high-throughput Yeast Two Hybrid screen (see Figure S1 and S2).

The total number of interactions consists of physical interactions, genetic interactions and other protein interactions (no interaction type or ambiguous annotations).

With about 180,000 interactions the by far largest eukaryotic interaction network is available for *S. cerevisiae*. The majority of interactions are genetic interactions. When we only consider physical interactions the *S. cerevisiae* interaction network is still the largest. Especially in comparison with the second largest protein interaction network from *H. sapiens* it becomes clear how sparse the networks for the other species still are in current databases. The *H. sapiens* network has fewer physical interactions, but more than four times more genes in the network as compared to *S. cerevisiae*.

Furthermore, only the *S. cerevisiae* network consists of only one connected component. It has been estimated that the complete *S. cerevisiae* network has between 37,800 and 75,500 protein interactions [57]. Actually, 55,767 physical interactions are contained in iRefIndex for *S. cerevisiae*. Therefore, for the following, we assume that the *S. cerevisiae* network is almost complete and, thus, we use the *S. cerevisiae* network to evaluate the performance of a protein interaction transfer.

It can be expected that more complex organisms also have a more complex network. The number of genes (and maybe also the number of proteins) is not dramatically different and, thus, most likely the number of interactions is different. Therefore, the extremely small coverage of even the best investigated model organisms is apparent. For all other non-model organisms the number of available interactions are neglegible.

### Interaction transfer

#### Experimental settings

We transfer interactions from all eukaryotic species with interaction data used in this study to *S. cerevisiae* to train our models. The *S. cerevisiae* interaction network is assumed to be almost complete and possible false negatives in the gold standard are ignored. True positives are defined as transferred interactions, which can be found in the *S. cerevisiae* network, and false positives as transferred interactions, which cannot be found in the network.

Three experimental settings are considered to evaluate our approach:

#### All interactions setting *(AllI)*

All interactions are transferred to *S. cerevisiae* and only the occurrence of the transferred interactions in the gold standard is checked.

#### Physical interactions setting *(PhyI)*

Only physical interactions are transferred to *S. cerevisiae* and in addition to the occurrence of the interactions also the agreement of the interaction type is checked.

#### Genetic interactions setting *(GenI)*

The same as the previous setting, but with genetic interactions.

In total 19,785 interactions from all eukaryotic species considered in this study can be transferred to *S. cerevisiae*. For *AllI* 4,745 interactions can be found in the gold standard and the other 15,040 interactions are used as negative set. The physical, *PhyI*, setting consists of 1,019 correctly transferred interactions and 8,174 incorrectly transferred interactions. The genetic, *GenI*, setting consists of 901 correctly and 5,300 incorrectly transferred interactions. The remaining 4,391 transferred interactions have an unknown, other, or an ambiguous interaction type.

The features are modeled for the protein pairs involved in the transfer. In total four proteins are considered for each transfer (two proteins from the source network and two proteins from the target network). The features are defined between the different protein pairings in the target network, in the source network and between the orthologs. In total 20 different features types are modeled where for the features used for the orthologs one feature for each of the two orthologs pairs involved in the transfer is created. E.g. for the global GO similarity one feature is modeled between the interaction partners in the source network, one feature is modeled between the interaction partners in the target network and two features are modeled between the orthologs involved in the transfer. For the gene expression feature the compiled gene expression experiment set from [58] which includes normalized intensity values from different cellular states and biological conditions is used.

Six feature sets are constructed for the training of the Random-Forest-Filter (*RFF*) in order to compare the performance and to estimate the feature contribution. This includes two main sets, one in which all features are considered and one setting where only features are used which can be assumed to be available for most of the species. Hence, features containing information about the network structure and the gene expression correlation are excluded in the reduced feature set. The other four feature sets (Network, Gene Ontology, General and Orthologs) consists only of the features from the respective category. In Table 2 the composition of the different feature sets and protein pairings is given.

**Table 2:** Feature set configuration.

	Pairing					Feature set			
	Pairing Source network (S)	Target network (T)	Ortholog (O)	Network	GO	General	Orthologs	Reduced	Full
Network overlap	x	x		x					x
GO Network	x	x		x					x
GO Global	x	x	x		x			x	x
GO (B) similarity (GO Biological process)	x	x	x		x			x	x
GO (C) similarity (GO Cellular component)	x	x	x		x			x	x
GO (M) similarity (GO Molecular function)	x	x	x		x			x	x
Source interaction database	x					x		x	x
Edge support	x					x		x	x
Source interaction type	x					x		x	x
Total support		x				x		x	x
Gene expression correlation		x				x			x
Sequence identity			x				x	x	x
Token similarity			x				x	x	x
Domain similarity			x				x	x	x
KEGG pathway score			x				x	x	x
Ortholog source			x				x	x	x
Ortholog score			x				x	x	x
Ortholog support			x				x	x	x
Transitive orthology			x				x	x	x
Phylogenetic distance			x				x	x	x

The table lists the features in the categories ”Network”, ”GO”, ”General” and ”Ortholog” and the protein pairings (proteins in the target network, proteins in the source network, orthologs). Also the configuration of the full (target network needs to be available) and the reduced feature set (used in real prediction filtering) is shown. For example the feature *Global GO* is modeled employing the interaction partners in the source network, in the target network and between the orthologs. Furthermore, the feature is included in the GO, the reduced and the full feature set.

#### Direct protein interaction transfer

**Figure 1:**
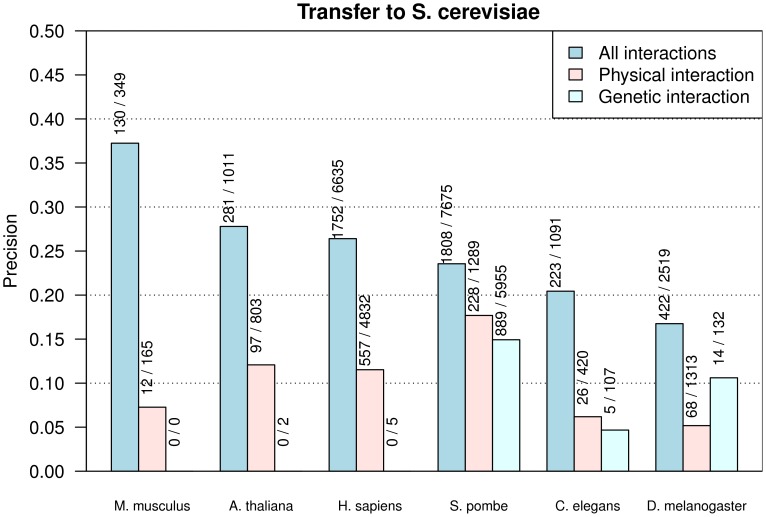
Precisions of a direct interaction transfer to *S.* *cerevisiae* for the different experimental settings. We show six eukaryotic species having the largest number of interactions. For the all interaction setting (*allI*) only the occurrence of a transferred interaction in the *S. cerevisiae* network is required, whereas for the genetic (*GenI*) and physical (*PhyI*) interaction setting also the exact type of the transferred interaction is checked. In addition to the precisions, the number of total transferred interactions and consistent interactions for each species and type is shown on top of the corresponding bar. Most interactions can be transferred from *S. pombe* to *S. cerevisiae*. There the transfer precision is highest for physical and genetic interactions. For the *allI* setting the highest transfer precision is observed for *M. musculus* to *S. cerevisiae*. This is due to the small number of interactions, which are mostly involved in conserved biological processes like DNA replication and chromosome organization.

Using the previously described interaction database and ortholog mappings, interactions are directly transferred to *S. cerevisiae*. In Figure 1 the precisions of the interaction transfers from six interaction networks using the previously introduced experimental settings are shown.

We use orthologs from the well established cluster based ortholog detection approaches InParanoid, OMA and HomologGene. Orthologs from these databases result in higher transfer consistencies than orthologs from tree based approaches like EnsemblCompara[59] (see Figure S3).

The overall precision of an interaction transfer from the different species to *S. cerevisiae* for *AllI* is 0.24, whereas for *GenI* and *PhyI* the transfer precision is only 0.11 and 0.15, respectively. With 4,745, 1,019 and 901 correctly transferred interactions, 3%, 2% and 1% of the *S. cerevisiae* network can be predicted for the respective experimental settings *AllI*, *PhyI* and *GenI*. The highest transfer precision of physical and genetic interactions can be achieved with a transfer from *S. pombe* (the phylogenetically closest species in our tree with experimentally validated interaction data).

Given complete interaction data for all species it would be expected that the highest precision would be achieved with a transfer from the phylogenetic closest species. But since the interaction data is incomplete and interologs of *S. cerevisiae* might be used as prior knowledge for the interaction discovery, some phylogenetically more distant species show higher interaction transfer precisions than phylogenetically closer species. Most notable is the performance of a transfer from *M. musculus* to *S. cerevisiae* with an unusually high precision of 0.36 in the *AllI* setting. A GO overrepresentation analysis (DAVID [60]) of the proteins involved in the transfer from *M. musculus* to *S. cerevisiae* exhibits that some highly conserved biological processes are overrepresented (like DNA-dependent DNA replication, pre-replicative complex assembly, DNA replication initiation and chromosome organization), which might explain the high precision of the interaction transfer. By looking at the transfer precisions for each biological process it can be seen that for these overrepresented biological processes the transfer precision from *M. musculus* to *S. cerevisiae* is almost the same as the transfer precision from *H. sapiens* to *S. cerevisiae*. E.g. 102 transferred interactions from *H. sapiens* to *S. cerevisiae* are associated with the biological process DNA-dependent DNA replication from which 64 are consistent, for the pre-replicative complex assembly process 30 out of 43 and for the DNA replication initiation process 31 out of 45 are consistent.

For phylogenetically distant species ortholog clusters consist of more than two genes which results in 1:n or even n:m mappings. Thus, with a direct transfer a single source interaction can be inferred between different genes in the target network. For *H. sapiens* and *S. cerevisiae* are for example on average 1.9 *H. sapiens* genes and 1.18 *S. cerevisiae* genes in one cluster, whereas for *H. sapiens* and *M. musculus* the cluster contain 1.05 and 1.01 genes, respectively.

#### Transfer filter

We train our Random-Forest-Filters (*RFF*) to score directly transferred interactions and to identify possible conservations.

**Figure 2:**
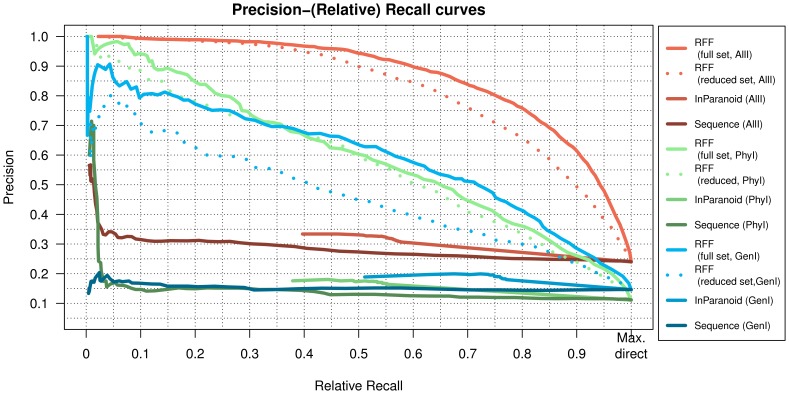
Precision - (relative) Recall curves. Precision - (relative)Recall curves for the *RFF* (Random-Forest-Filter) trained with the reduced feature set, the full feature set and different experimental setting (only physical interactions (*PhyI*), only genetic interactions (*GenI*) and all interactions (*AllI*)) using 10-fold cross validation. Interactions are transferred from all eukaryotic species with interaction data to *S. cerevisiae* and filtered with the respective approaches. In addition, the precision and relative recall is given for a simple sequence similarity filter and a filter based on the InParanoid ortholog bootstrap score. The *RFF* for *AllI* trained with the full (red) and reduced (red dotted) feature set perform best. The reduced feature set performs somewhat worse than the full feature set. For the more strict *PhyI* and *GenI* settings in which also the type of an interaction is transferred, the performance drops in comparison to *AllI*. By comparing the different feature sets it can be seen that for physical interactions (green, green dotted) almost the same performance for the full and reduced feature set can be reached, whereas for genetic interactions (blue, blue dotted) a clear difference in the performance can be observed. But again, for these two settings a huge improvement of *RFF* to the baseline filters based on sequence similarity) and ortholog scores can be observed.

In Figure 2 the precision-(relative) recall curves of the Random-Forest-Filters (*RFF*) trained with the full and reduced feature sets and the three experimental settings *AllI*, *PhyI* and *GenI* using a 10-fold cross validation are shown. A simple interaction filter using the harmonic sequence similarity between the orthologs and a filter based on the InParanoid ortholog bootstrap score are evaluated as baseline comparisons.

The *RFF* trained with the full feature set in the *AllI* setting achieves the highest 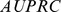 score of 0.86 and an 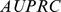 score of 0.82 with the reduced feature set. When in addition to the occurrence of an interaction also an interaction type agreement is required, the performance drops significantly. Physical interactions can be classified with an 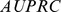 score of 0.60 and of 0.58 with the *RFF* trained with the full and reduced feature set, respectively. Genetic interactions can be classified with 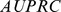 score of 0.60 and 0.48.

Using a maximum InParanoid ortholog bootstrap score of 1.00, a transfer precision of 0.33 for *AllI* can be reached resulting in an 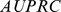 of 0.30. For physical and genetic interactions the precision of a direct transfer can barely be improved resulting in an 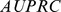 of 0.15 and 0.18, respectively.

A high threshold has to be used for the sequence similarity filter in order to increase the transfer precision resulting in a low 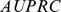 score of 0.28 for *AllI*. Even lower are the 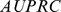
s for *PhyI* and *GenI*. This can be explained with the low sequence similarities of the orthologs used for the transfer, which ranges between 33% and 38% on average for the different species. For the full feature set the *RFF* for *AllI* yields a precision of 0.85 and a relative recall of 0.69 (regular recall of 0.02) with a typical score threshold of 0.5. With the same score threshold for *PhyI* a precision of 0.72 and a relative recall of 0.33 (regular recall of 0.01) can be reached, whereas for *GenI* a slightly lower precision of 0.68, but a higher relative recall of 0.40 is observed (0.003 regular recall).

In general, the predictors for *AllI* achieve a better performance than the predictors for the more strict setting in which also the interaction type has been transferred and predicted. This is plausible as for *AllI* the gold standard is larger and as with a direct transfer a consistency of 25% can be reached already. For the different feature sets (full and reduced) a small drop in the *AllI* and *PhyI* setting and a large drop for the *GenI* setting is observed.

In the following we show examples of transferred physical interactions which receive high and low score values by *RFF*. On one hand, the transferred interaction between LST8 and TOR2 from WAT1 and TOR2 (in *S. pombe*) and also the transferred interaction between SMX3 and LSM5 from SmF and CG6610 (in *D. melanogaster*) get a comparable high score of 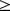 0.90. For the first, but not for the second example also an interaction is known between the orthologs in *S. cerevisiae*. But for the second example, both orthologs (SMX3 and LSM5) carry the Sm domain and the interaction between orthologs of SmF and CG6610 have been found in *S. pombe* and *H. sapiens*, which suggests that SMX3 and LSM5 indeed interact, but that they are not included in the *S. cerevisiae* gold standard. On the other hand, the transferred interaction between CRZ1 and HAT2 from Sp3 and RBBP4 (in *H. sapiens*, identified within a low-throughput study[61]) and the transferred interaction between ARP6 and RPS1A from Actr13E and RpS3A (in *D. melanogaster* which was identified in a Yeast Two Hybrid screen [8]) gets a score of 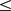 0.05. Both transferred interactions are not in the *S. cerevisiae* gold standard, therefore, they are filtered correctly. Due to the low-throughput experiment, which was used to discover the interaction between Sp3 and RBBP4 it can be assumed that this interaction indeed exists for *H. sapiens*, but not in *S. cerevisiae*. In contrast, the interaction between Actr13E and RpS3A could also be false positive due to the high-throughput Yeast Two Hybrid screen which was used to identify the interaction in *D. melanogaster*. In Figure S4 (Supporting Information) the transferred interactions together with their assigned *RFF* scores and their feature values in comparison to the feature distributions of correctly and incorrectly transferred interactions are shown.

#### Feature impact

**Figure 3:**
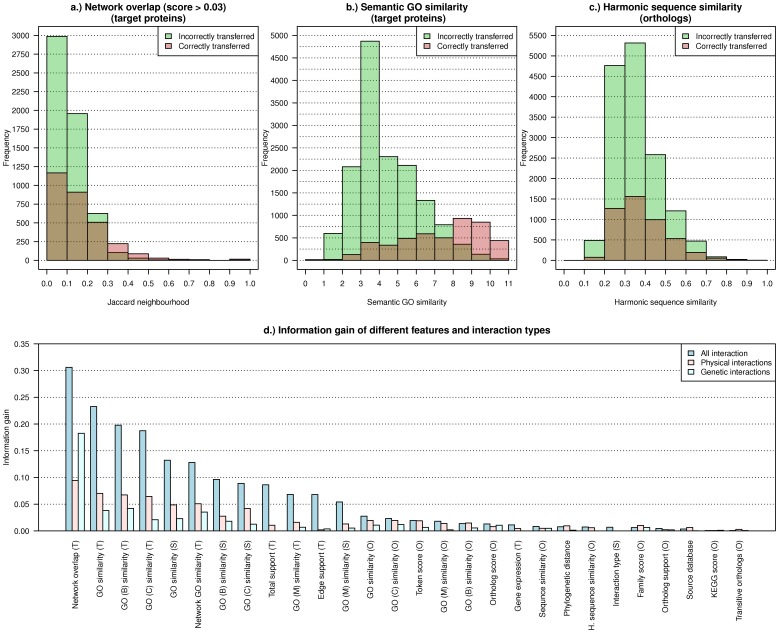
Feature impact. Histogram of the score values for correctly (red) and incorrectly (green) transferred interactions (without interaction type) for the features: **a.)** Network overlap, **b.)** Semantic GO similarity and **c.)** Harmonic sequence similarity. **d.)** Information Gain of the individual features and experimental settings. For ortholog protein features the average Information Gain of the two orthologous partners is shown. For the features **a.)** Network overlap and especially for **b.)** Semantic GO similarity a different distribution for correctly and incorrectly transferred interactions can be observed resulting in a large Information Gain of these features. In contrast, for the harmonic sequence similarity feature only a small difference in the distributions can be observed, which explains the small Information Gain and the filter performance based only on sequence similarity.

To estimate the contribution of each feature to the performance of *RFF*, the Information Gain (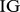) is computed for the different experimental settings (Figure 3 d). The 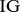 for the different features differs among the experimental settings, but the sorting of the features according to their 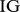 value is similar. The strongest feature is the network overlap in the target network (Network overlap (T)). But also the GO features yield a high 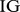. The combined GO features have higher 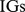 than the category-wise GO features for biological processes, cellular components and molecular functions. This can be explained by the fact that more GO terms are considered for the global semantic GO similarity, so less often a missing value indicator is assigned. From the individual GO term types, the biological processes category has the highest 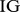. Biological processes have also been identified by [20] as a strong feature to define thresholds for an interaction transfer filter. From the Ortholog features the synonym similarity (token score) and the ortholog score feature contributes most to the prediction.

In contrast, the gene expression correlation, which was used in other studies for the prediction of protein interaction, has a rather low 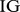. For the two features with highest 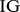 (Network overlap and GO similarity in the target network) also the score distributions of correctly and incorrectly transferred interactions for *AllI* are shown in Figure 3. Clearly, the fraction of correctly to incorrectly transferred interactions increases with the score value for these two features. For a feature like the harmonic sequence similarity, which has a low 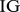, only a small difference in the characteristics of the distribution can be observed, which explains the weak performance of filters based on sequence similarity.

In Table 3 the performance of the different individual feature sets (Network, Gene Ontology, General, Ortholog, Reduced set and Full set) is summarized in addition to the filters based on the sequence similarity and the InParanoid bootstrap score. For the GO features the highest feature category-wise 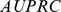 score can be reached for *AllI* and *PhyI*. For *PhyI* a similar 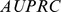 score can be achieved with the ortholog features. Using a combination of all introduced features an up to 0.08 higher 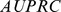 score can be obtained for the different settings. For *GenI* the highest category-wise 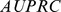 score can be reached with the network features, which is also higher than the score for the reduced feature set. This explains the performance drop for the reduced feature set for this *GenI* setting.

**Table 3:** Result details for the transfer to *S. cerevisiae.*

Method	Experimental	Feature	AUPRC	AUROC
	setting	set		
RFF	All	Full	0.86	0.94
RFF	All	Reduced	0.82	0.91
RFF	All	Network	0.79	0.90
RFF	All	GO	0.79	0.89
RFF	All	Ortholog	0.62	0.82
RFF	All	General	0.50	0.68
InParanoid	All	-	0.30	0.59
Sequence	All	-	0.28	0.55
RFF	Physical	Full	0.60	0.89
RFF	Physical	Reduced	0.58	0.88
RFF	Physical	GO	0.50	0.85
RFF	Physical	Network	0.42	0.84
RFF	Physical	Ortholog	0.48	0.82
RFF	Physical	General	0.19	0.62
InParanoid	Physical	-	0.15	0.61
Sequence	Physical	-	0.14	0.55
RFF	Genetic	Full	0.60	0.87
RFF	Genetic	Reduced	0.47	0.82
RFF	Genetic	Network	0.51	0.86
RFF	Genetic	GO	0.45	0.80
RFF	Genetic	Ortholog	0.35	0.75
RFF	Genetic	General	0.19	0.53
InParanoid	Genetic	-	0.18	0.60
Sequence	Genetic	-	0.15	0.51

10-fold cross validation results of the *RFF* (Random-Forest-Filter) trained with different feature sets, the InParanoid ortholog filter and the sequence similarity filter for different experimental settings. Interactions are transferred from all eukaryotic species with interaction data to *S. cerevisiae*.

For each experimental setting and feature set the area under precision recall curve (AUPRC) and the area under the receiver operating characteristic curve (AUROC) are computed. From the individual feature sets the *RFF* trained with the GO and Network feature set perform best for the *AllI* and *GenI* setting. Whereas for physical interactions the performance for the Network features are lower than for the GO and Ortholog feature set.

#### Generalizability

A general transfer approach should be able to achieve a similar performance for the interaction transfer to other species. Since the interaction networks for other species are currently too sparse (see Table 1) *RFFs* can not be learned and evaluated for individual species except for *S. cerevisiae*. Therefore, we investigate the applicability of the *RFF* fitted for the interaction transfer to *S. cerevisiae* for the transfer of interactions to other eukaryotic species. It has to be expected that:

1. the *RFF* scores transferred interactions between phylogenetically closer species higher than transferred interactions between phylogenetically distant species,
2. according to their importance, the ranking of features is similar for the interaction transfer to different species even though the networks are to incomplete to train a model and
3. that a comparable performance with competing transfer approaches should be achieved when the *RFF* is applied for the transfer of interactions to other species.
4. In the following we investigate these three points.

#### Transfer scores

**Figure 4:**
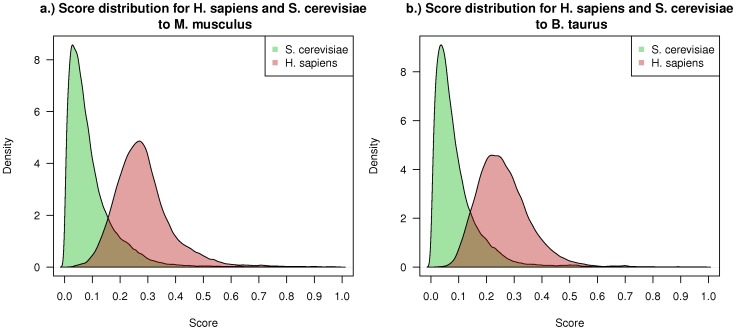
Score distributions. Score distributions for transferred interactions with *RFF* from *S. cerevisiae* and *H. sapiens* to the two target species (**a.**) *M. musculus* and (**b.)** *B. taurus*. Transferred interactions from *S. cerevisiae* have significantly lower score values than transferred interactions from *H. sapiens* to both target species. With a low score threshold of 0.2 almost all interactions from *H. sapiens* will be transferred to the two target species whereas a huge fraction of the transferred *S. cerevisiae* interactions is filtered out.

We use the *RFF* with the reduced feature set trained with transferred interactions to *S. cerevisiae* to transfer protein interactions from the two largest interaction networks *H. sapiens* and *S. cerevisiae* to both *M. musculus* and *B. taurus* and analysed the score distributions. For physical and genetic interactions in the source network, the predictor trained with the respective interaction type (*PhyI* and *GenI*) is used and for interactions with a different type the predictor trained with all data is applied (*AllI*). As expected, the scores for transferred interactions from the phylogenetically closer species, in this case *H. sapiens*, are higher than the scores from the more distant species as shown in Figure 4. The score distribution of transferred interactions from *S. cerevisiae* to *M. musculus* and *B. taurus* are very similar with a median score of 0.07 for both distributions. This is comparable to the transfer of interactions to *S. cerevisiae*, where a median score between 0.03 and 0.09 can be observed (see Figure S5). In comparison, for the transfer of interactions between phylogenetically closer species, a median score of 0.27 and 0.25 can be observed for the transfer of interactions from *H. sapiens* to *M. musculus* and *B. taurus*, respectively. Thus, as expected with higher score thresholds more interactions can be transferred from *H. sapiens* to *M. musculus* as compared to *H. sapiens* to *B. taurus*. On the other hand, from *S. cerevisiae* almost the same number of interactions is transferred to the two species *B. taurus* and *M. musculus* with different score thresholds.

#### Cross-species feature importances ranking

We transfer all available interactions to *S. cerevisiae*, *H. sapiens*, *D. melanogaster*, *S. pombe* and *C. elegans* and compute the Information Gain (IG) of each modeled feature given the observed consistently and inconsistently transferred interactions for the respective species. We observe that the similarity of the feature ranking decreases with the IG i.e. that those features which are important for the classifier are consistently ranked high and the ranking of those feature which are not that beneficial to our classifier differ more. For example the Network overlap feature is ranked first for all considered species expect for *C. elegans*. Also the feature which models the GO Similarity between the target interactions is ranked second by all considered species expect for *C. elegans*. In Figure S6 in the Supporting Information we show the ranking of the features according to their IG for the different species. As reference we use the ten features with highest IG for the interaction transfer to *S. cerevisiae*.

#### Comparison with other interaction transfer methods

Most protein interaction transfer methods predict interologs for *H. sapiens* and, in addition, quite many experimentally validated interactions are available for human. Therefore, this network is chosen to evaluate the intersections of predicted protein interactions from different data sets and a set of experimentally discovered physical protein interactions.

Transferred interactions from *InteroPORC* [21], the *STRING* database [62], *InterologFinder* [24], *BIPS:BIANA* [25] and interactions predicted with *RFF* are used for the comparison. In order to compare the sets, the protein identifiers are mapped to UniProt/Swissprot identifiers. The following prediction sets are constructed using the publicly available transferred networks from the considered approaches for *H. sapiens*:

#### *STRING(1)*

only high confidence interactions with at least one evidence of an interaction transfer from another species (interactions with a combined score below 0.7 are excluded);

#### *STRING(2)*

The combined score of *STRING* incorporates evidence from many sources including experimental knowledge for the respective species (direct evidence). Thus, transferred interactions with also direct evidence are scored higher, which biases the *STRING* set for this comparison. Therefore, an additional *STRING* interaction set is created where the combined score is recomputed without the scores for the direct evidence from databases, experiments and text-mining using the equation for the combined score [30]. Again for this set a combined score threshold of 0.7 is used to filter interactions.

#### *InteroPORC*

all transferred interactions;

#### *InterologFinder*

15,795 transferred interactions with highest score (the score threshold is set so that the same number of interaction as *STRING(2)* are predicted);

#### *BIPS;BIANA*

all transferred interactions in the online available precomputed prediction set with domain interactions or shared GO terms;

#### *RFF*

The *RFF* for physical source interactions (*PhyI*) trained with the reduced feature set and with transferred interactions to *S. cerevisiae* is used. All transferred interactions with a score 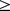 0.18 for the transfer to *H. sapiens* from all species considered in the study are used. The score value is experimentally chosen to yield roughly the same number of transferred interactions as *STRING(2)*.

In the entire *InteroPORC* prediction set 17,111 physical interactions and in the selected *BIPS:BIANA* set 7,073 interactions are included. With 28,155 links between proteins the interaction set from the *STRING(1)* is the largest, the *STRING(2)* set is only slightly larger (15,795 interactions) than the set from *RFF* which includes 14,634 predicted physical interactions. 35,628 experimentally validated physical interactions are taken from iRefIndex (7,784 interactions are excluded because the proteins are only mappable to UniProt/TrEMBL).

**Figure 5:**
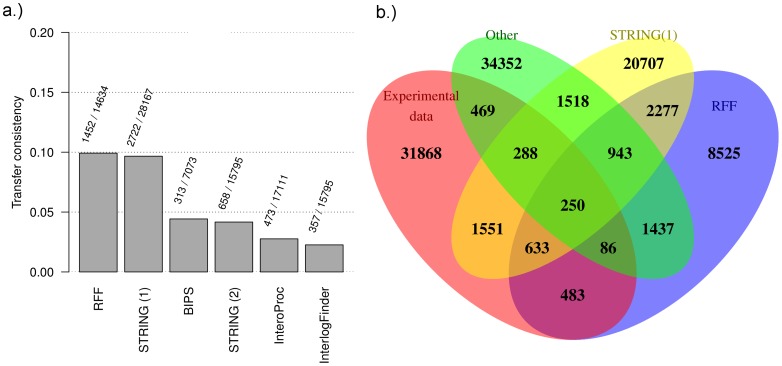
Method comparison. Comparison of interaction transfer sets from various methods for *H. sapiens* with known *H. sapiens* interactions from iRefIndex. We compare interaction sets from *STRING* [62], *InteroPORC* [21], *InterologFinder* [24], *BIPS:BIANA* [25] and our Random-Forest-Filter (*RFF*). From the *STRING* database only interactions with interaction transfer information from other species and a combined score over 0.7 are included (*STRING(1)*). The combined score uses information from all information sources including knowledge on experimental interactions for the respective species (direct evidence). Therefore, an additional interaction set is created where the combined *STRING* score is recomputed excluding the scores from the direct evidence of databases, experiments and text-mining (*STRING(2)*). In general, the intersections between the different sets and the known interactions are small. **a.)** With the *RFF* and with *STRING(1)* 10% of the predicted interactions can be found in the experimental data. The modified *STRING(2)* interaction set is 43% smaller and only 4% of the predicted interactions are consistent with the experimental data showing a clear performance advantage of the *RFF* for species with no experimentally determined interactions. **b.)** We compare the interaction sets of *RFF*, *STRING(1)*, a combined set of unique interactions from *InteroPORC*, *InterologFinder* and *BIPS:BIANA* and a set of known *H. sapiens* interactions. With the *RFF* 42% of predicted interactions can also be found in one of the other sets.

In Figure 5 the consistency with experimentally validated interactions (**a.**) and the intersections between different *H. sapiens* protein interaction sets are shown (**b.**).

In general, the intersections between the sets are small. The highest consistency of 10% between the predicted interaction sets and the experimental set can be reached with the *RFF* and with the *STRING(1)* interaction set.

From the *STRING(2)* and *BIPS:BIANA* interaction set 4% and from the *InteroPORC* and *InterologFinder* around 3% of the predicted interactions are consistent with the experimental data. In total 42% of predicted interactions with the *RFF* can be found in at least one other set whereas for the *STRING(1)* set only 26%, for the *BIPS:BIANA* set 18%, for the *InteroPORC* set 17% and for the *InterologFinder* set 10% can be found in another interaction set. Besides *BIPS:BIANA* all methods transfer interactions from all available interactions in public available databases. But *BIPS:BIANA* explicitly excludes interactions from Tandem Affinity Purification experiments which explains the rather small interaction set. In comparison to *STRING(2)*, *BIPS:BIANA*, *InteroPORC* and *InterologFinder* a clear performance gain of our *RFF* approach can be observed. Furthermore, *RFF* cannot be outperformed by *STRING(1)* even with the integration of experimental knowledge (which is not available for most species) via the combined score. Thus, for species without experimental knowledge but also for model organisms with experimental protein interactions a performance advantage of our approach in comparison to *STRING* can be expected.

### Enriched protein interaction networks

As shown above via the comparison with other state-of-the-art method our *RFF* approach has a decent performance for the transfer of interactions to species without experimental interaction data. Therefore, we use our approach to obtain as comprehensive as possible interaction networks for various eukaryotic species. For this we use all available experimental interaction data for all 83 eukaryotic species for the transfer to all other eukaryotic species whenever ortholog mappings of appropriate quality are available. We employ three *RFFs* trained on *S. cerevisiae*: *RFF, PhyI* for physical source interactions, *RFF, GenI* for genetic source interactions and *RFF, AllI* for interactions for the remaining interactions including interactions without annotated interaction type. The same score threshold of 0.18 is used for all models.

**Figure 6:**
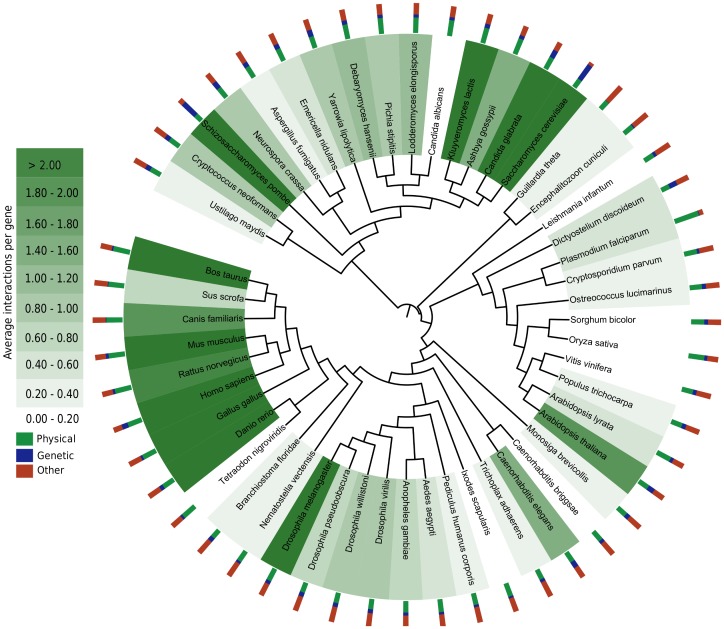
Enriched interactome. The interactomes of 83 eukaryotic species can be increased from currently 321,808 interactions to 1,076,996 pair-wise interactions using a score threshold of 0.18 and the *RFFs* with reduced feature set. In the figure the enriched protein interactomes are shown for all species where at least GO annotations for half of the genes are available. Interactions are transferred from all eukaryotic species to all other species with available ortholog mappings. The color of the species nodes indicates the average number of interactions per gene and the associated bar chart indicates the fraction of physical interactions (green), genetic interactions (blue) and other interaction types (red) in the enriched interaction networks for the respective species. For species with rich annotation information including *M. musculus* and *B. taurus* over 40,000 interactions can be transferred resulting in an average number of interactions per gene larger than 2. For species with sparse annotation information and few ortholog references to UniProt only a small number of interactions can be transferred. For example for the plants *O. sativa* and *V. vinifera* only 0.35 interactions per gene on average can be obtained.

With *direct* interaction transfer the interactome of 83 eukaryotic species can be extended from currently 321,808 interactions to 5,751,775 interactions. With the *RFF* 1,248,609 pair-wise interactions can be transferred (i.e. more than 78,% of transferred interactions are filtered out as possible false positive). An overview of the resulting interactomes is shown in Figure 6 using the Interactive Tree Of Life [63] (only species are shown for which at least 50% of the genes have associated GO annotations). For higher vertebrates of interest such as the farm animals *B. taurus*, *M. musculus* and *G. gallus* each interaction network can be enriched with over 40,000 interactions. After that, the resulting interactomes have a decent coverage of more than 2 interactions per gene on average. Still, with our method for some species only few interactions can be transferred. Examples are plants like *O. sativa* or *V. vinifera* with an average of 0.35 interactions per gene. The reason for the low coverage in these cases is the small number of available orthologs in the ortholog databases.

It is clear that for the large scale interaction transfer with our *RFF* method the limitations are the availability of ortholog relations, of mappings of the orthologs to UniProt entries and of annotations of the UniProt entries. This implies that for some species only few interactions can be transferred. Of course, *RFF* will profit from the expected improvements of protein annotations, ortholog mappings and further experimental protein interactions.

**Figure 7:**
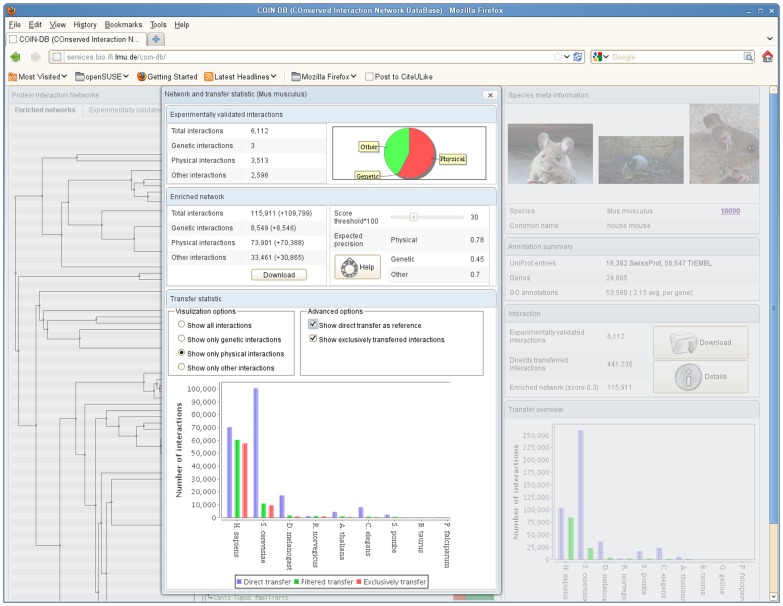
COIN-DB web interface. Screenshot of the web interface for the transferred and scored protein interactions. Transferred and experimentally validated interactions can be downloaded for 83 eukaryotic species for user specified score thresholds. For species of interest the transfer profiles can be inspected in detail including the number of interactions (of the different interaction types) and the number of uniquely transferred interactions, and the expected performance of the transfer.

The transferred interaction networks for the 83 species are available on our web service and can be inspected and downloaded. The user can specify score thresholds corresponding to the expected transfer precision of our models. The database will be frequently updated to incorporate newly available experimental interactions and updates of protein annotations and orthologs for more species. In Figure 7 the web interface including the ‘transfer statistics view’ for *M. musculus* is outlined as an example.

## Discussion and Conclusions

Years after high-throughput screening techniques for the identification of protein interactions were introduced most interaction data still is available for only a few model organisms, in particular for *S. cerevisiae*. Transferring protein interactions works best between phylogenetically close species, but already between the two yeast species *S. pombe* and *S. cerevisiae* only a consistency of 36% for transferred physical interactions can be observed. The transfer consistency between more distant species is of course much lower. The transfer consistency is also lower for genetic interactions between the two yeasts, which might be due to the incompleteness of the *S. cerevisiae* genetic interaction network.

We observed that for only 3% of the *S. cerevisiae* interactions evidence of conservation between orthologs in different species could be found. In order to improve the transfer quality and to be able to also consider interactions from phylogenetically distant species, e.g. from *S. cerevisiae* to *M. musculus*, we introduced a new method using Random Forests (Random-Forest-Filter *RFF*) to score and filter transferred interactions.

We trained the models with transferred interaction data from eukaryotic organisms to *S. cerevisiae*. We did the training on yeast, as the *S. cerevisiae* network is currently the largest eukaryotic interaction network and for most of the proteins in the network curated functional annotations are available. We evaluated the models with different feature sets and experimental settings and compared the models with commonly applied filter approaches e.g. using the sequence similarity and the InParanoid bootstrap score. We showed that for the task of transferring interactions to *S. cerevisiae* our approach performs better than commonly applied filter approaches. Based on these results we assume that the performance of the transfer to *S. cerevisiae* is a lower bound for the performance of the method for the transfer between phylogenetically closer species.

But still, our observed results are limited with respect to different aspects:

1. Possible false negatives in the *S. cerevisiae* network result in lower transfer consistencies, whereas false positives in the *S. cerevisiae* network may result in an overestimation of the consistency.
2. Our method makes use of interaction data from various sources like Yeast two Hybrid, or Tandem Affinity Purification and thus included measured-binary and measured-predicted binary interactions. We only address the interaction transfer on a general level and currently only consider binary-interactions. Our method will benefit from further discrimination of protein interactions e.g. discrimination between transient or permanent protein interactions, or the pre-identification of conserved protein complexes. And thus, stronger claims on the conservation rate and also a more complete interaction transfer will be possible.
3. Low-throughput experiments are commonly hypothesis-driven [4] and involve proteins of particular interest to the researcher performing the experiments. These low-throughput experiments can also be based on the observation that a conservation in a particular species exists, which could lead to an overestimation of the consistency and to overfitting.
4. The ortholog and protein annotations quality have a direct influence on our models. For example KEGG pathway information, or gene ontology and synonym annotations are themselves often inferred using homology information ( directly or indirectly). For example the KEGG databases transfers pathway information from well studied species based on manually defined ortholog groups. It is obvious that with solely transferred annotations our approach can not improve the prediction performance.
5. We fitted our model for the transfer to *S. cerevisiae* only. Due to these reasons, we can not give an accurate estimation on the performance for the protein interaction transfer to species except for *S. cerevisiae*.

But we could show that our approach can be applied for the transfer of interaction to species beyond *S. cerevisiae* as well. On one hand, we tested the generalizability of *RFF* with transferred interactions to *H. sapiens*, *M. musculus* and *B. taurus*. We showed that (as expected) transferred interactions from phylogenetically closer species get higher scores than transferred interactions from phylogenetic more distant species. Furthermore, we showed that those features which are most beneficial for the classification of interaction for the transfer to *S. cerevisiae* are also most beneficial for the classification of interactions for other species. On the other hand, we compared different protein interaction approaches. We showed for *H. sapiens* that with our approach the highest consistency of transferred interactions can be observed and that 42% of transferred interactions can be explained with high confidence relations extracted from *STRING*, *InteroPORC*, *InterologFinder*, *BIPS:BIANA* or the available experimental interactions. Furthermore, in an experimental setting where we recomputed the *STRING* combined edge score for *H. sapiens* to mimic a species without experimental knowledge, we showed that *RFF* predicts almost the same number of interaction as *STRING*, but with our approach more than twice as many interactions are consistent with the available experimental protein interaction network.

Therefore, we used *RFF* to transfer protein interactions to *83* eukaryotic species and we provide a web service for the download and investigation of these transferred interaction networks. Based on the above discussions, these predictions have to be used with care, but we are confident that our transferred networks are as comprehensive and also as accurate as currently possible.

### 

#### Availability

http://services.bio.ifi.lmu.de/coin-db/.

## Supporting Information

Figure S1
**Fraction of interactions derived from low and high-throughput studies.** Protein interactions for *S. cerevisiae*, *H. sapiens*, *D. melanogaster* and *S. pombe* from iRefIndex[33] classified into the categories: derived from low-throughput studies (detected in studies which report between 1 and 10 interactions), derived from mid-throughput studies (detected in studies which report between 10 and 100 interactions), derived from mid-high throughput (detected in studies which report between 100-1000 interactions) and derived from high-throughput studies (detected in studies which report 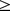
1000 interactions).(PDF)

Figure S2 **Interactions by detection method.** Protein interactions by detection method for *S. cerevisiae*, *H. sapiens*, *D. melanogaster* and *S. pombe* from iRefIndex[33].
(PDF)

Figure S3 **Direct interaction transfer to** ***S. cerevisiae*** **using different ortholog databases.** Transfer consistencies of a protein interaction transfer from *M. musculus*, *H. sapiens*, *S. pombe*, *C. elegans* and *D. melanogaster* to *S. cerevisiae* using orthologs from the databases OMA[45], InParanoid [46], HomoloGene [47], EnsemblCompara[59], TreeFam[64] and eggNog[65] for the all interaction setting (*allI*).
(PDF)

Figure S4 **Transfer examples.** Examples of transferred interactions which get high and low scores by *RFFs* including specific feature values for these interactions and the overall feature distribution (the scores are estimated via a cross-validation setting).
(PDF)

Figure S5 **Protein interaction scores for the transfer of interactions to** ***S. cerevisiae*** **.** The average transfer scores for an interaction transfer from *M. muscles*, *H. sapiens*, *S. pombe*, *A. thaliana*, *C. elegans* and *D. melanogaster* to *S. cerevisiae* using *RFFs* in a cross-validation setting.
(PDF)

Figure S6 **Information Gain feature ranking.** The feature importance ranking i.e. the ranking of features, is quite similar especially for the most important features, whereas the ranking of the less important feature varies more.
(PDF)

Table S1 **Data sources**. List of data sources used for this study.
(XLS)
