# Supplementary material for: Complementing the Eukaryotic Protein Interactome
Source: PLoS One. 2013 Jun 18;8(6):e66635. doi: 10.1371/journal.pone.0066635 (PMC3688968; doi:10.1371/journal.pone.0066635)
Supplement: Figure S1 — Fraction of interactions derived from low and high-throughput studies. Protein interactions for S. cerevisiae, H. sapiens, D. melanogaster and S. pombe from iRefIndex[33] classified into the categories: derived from low-throughput studies (detected in studies which report between 1 and 10 interactions), derived from mid-throughput studies (detected in studies which report between 10 and 100 interactions), derived from mid-high throughput (detected in studies which report between 100-1000 interactions) and derived from high-throughput studies (detected in studies which report 1000 interactions). (PDF) [file pone.0066635.s001.pdf]

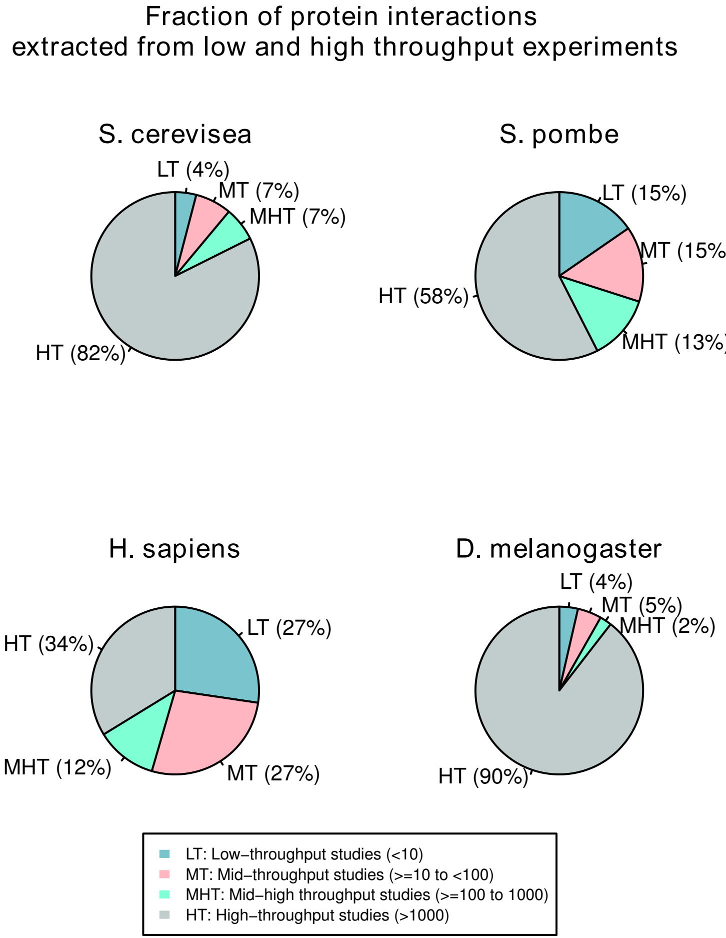

Fraction of protein interactions
extracted from low and high throughput experiments
S. cerevisea
LT (4%)
MT (7%)
MHT (7%)
HT (82%)
S. pombe
LT (15%)
MT (15%
MHT (13%
HT (58%)
H. sapiens
LT (27%)
MT (27%)
MHT (12%)
HT (34%)
D. melanogaster
LT (4%)
MT (5%)
MHT (2%)
HT (90%)
LT: Low−throughput studies (<10)
MT: Mid−throughput studies (>=10 to <100)
MHT: Mid−high throughput studies (>=100 to 1000)
HT: High−throughput studies (>1000)
