# Supplementary figures and images for: Complementing the Eukaryotic Protein Interactome
Source: PLoS One. 2013 Jun 18;8(6):e66635. doi: 10.1371/journal.pone.0066635 (PMC3688968; doi:10.1371/journal.pone.0066635)

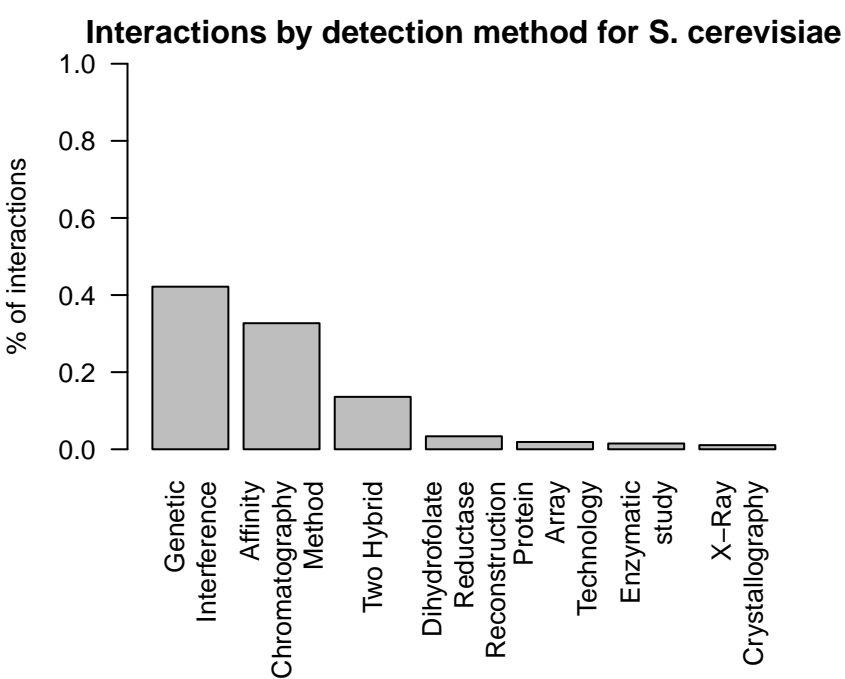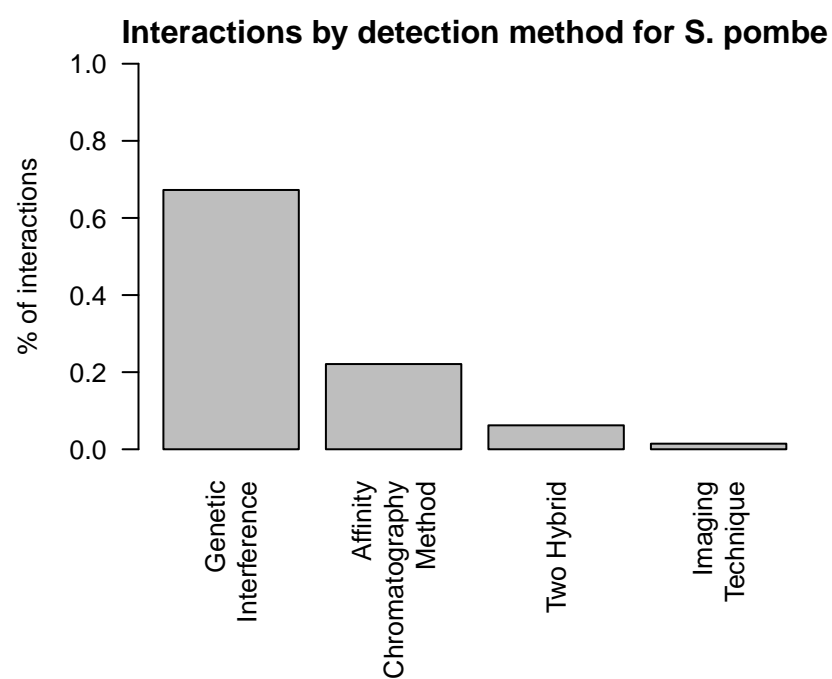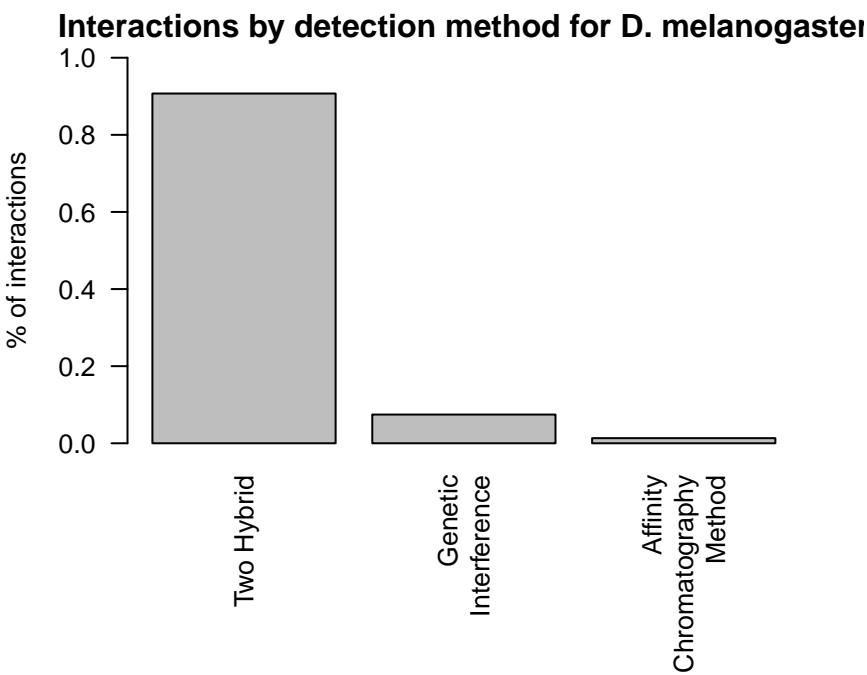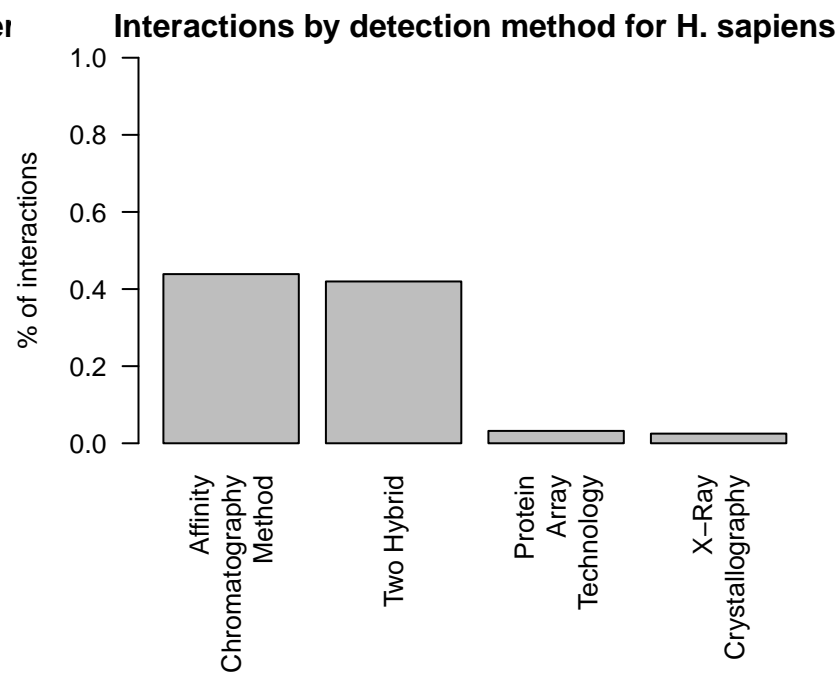

Supplement: Figure S2 — Interactions by detection method. Protein interactions by detection method for S. cerevisiae, H. sapiens, D. melanogaster and S. pombe from iRefIndex[33]. (PDF) [file pone.0066635.s002.pdf]

## Direct transfer to *S. cerevisiae* using different ortholog databases

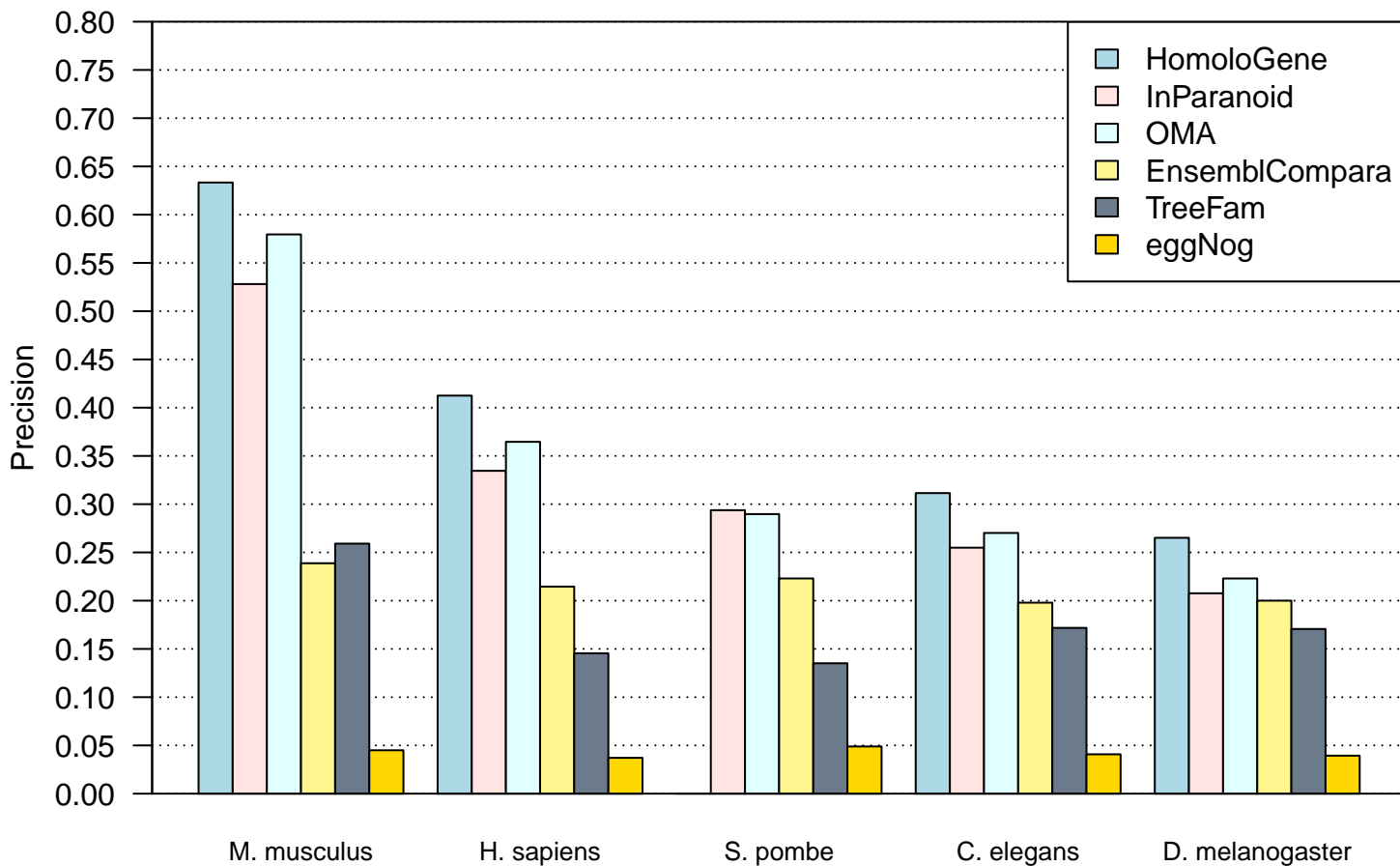

Supplement: Figure S3 — Direct interaction transfer to S. cerevisiae using different ortholog databases. Transfer consistencies of a protein interaction transfer from M. musculus, H. sapiens, S. pombe, C. elegans and D. melanogaster to S. cerevisiae using orthologs from the databases OMA[45], InParanoid [46], HomoloGene [47], EnsemblCompara[59], TreeFam[64] and eggNog[65] for the all interaction setting (allI). (PDF) [file pone.0066635.s003.pdf]

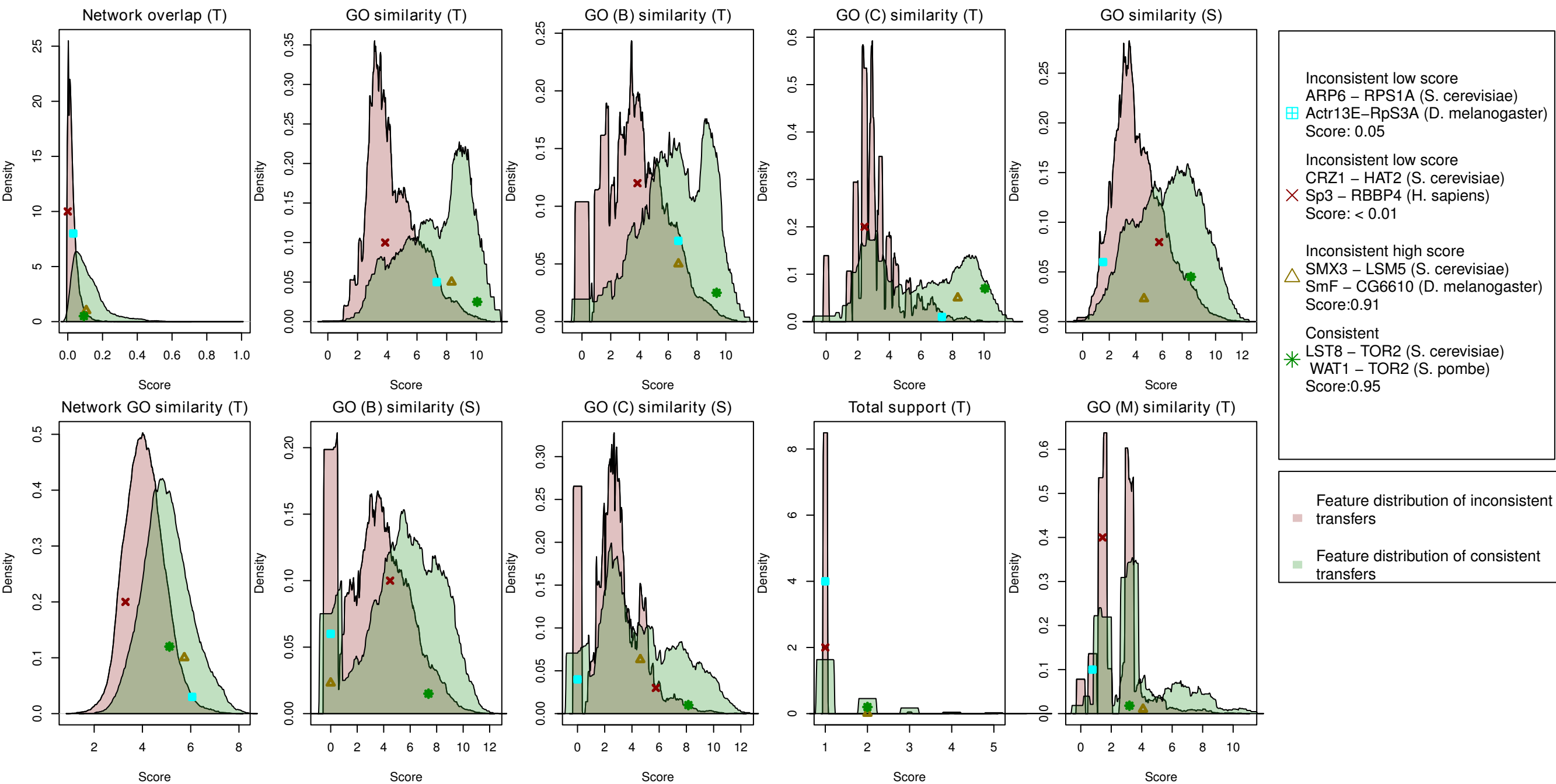

Supplement: Figure S4 — Transfer examples. Examples of transferred interactions which get high and low scores by RFFs including specific feature values for these interactions and the overall feature distribution (the scores are estimated via a cross-validation setting). (PDF) [file pone.0066635.s004.pdf]

## Cross-training Information Gain

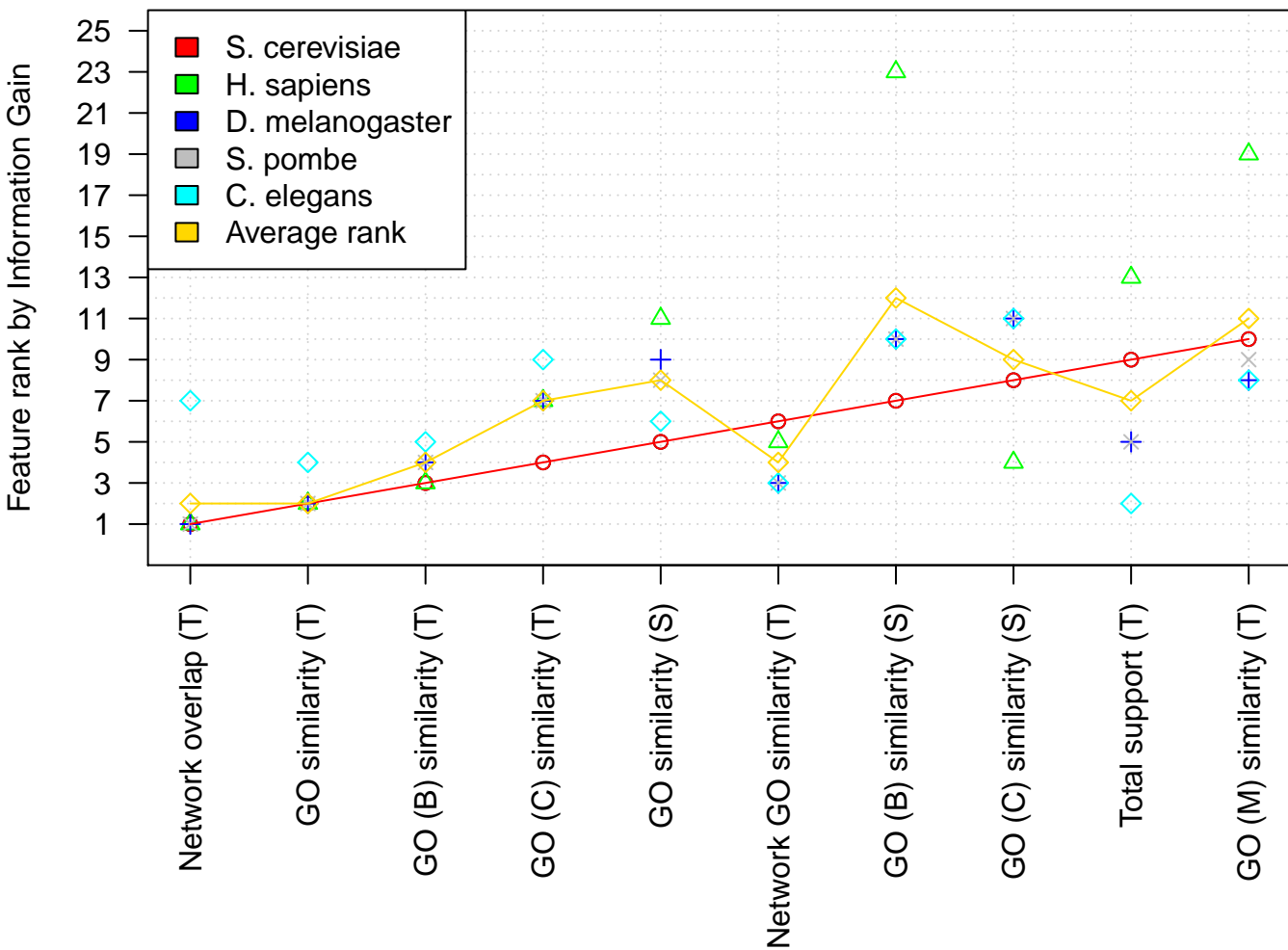

Supplement: Figure S6 — Information Gain feature ranking. The feature importance ranking i.e. the ranking of features, is quite similar especially for the most important features, whereas the ranking of the less important feature varies more. (PDF) [file pone.0066635.s006.pdf]
