# Supplementary material for: Complementing the Eukaryotic Protein Interactome
Source: PLoS One. 2013 Jun 18;8(6):e66635. doi: 10.1371/journal.pone.0066635 (PMC3688968; doi:10.1371/journal.pone.0066635)
Supplement: Figure S5 — Protein interaction scores for the transfer of interactions to S. cerevisiae . The average transfer scores for an interaction transfer from M. muscles, H. sapiens, S. pombe, A. thaliana, C. elegans and D. melanogaster to S. cerevisiae using RFFs in a cross-validation setting. (PDF) [file pone.0066635.s005.pdf]

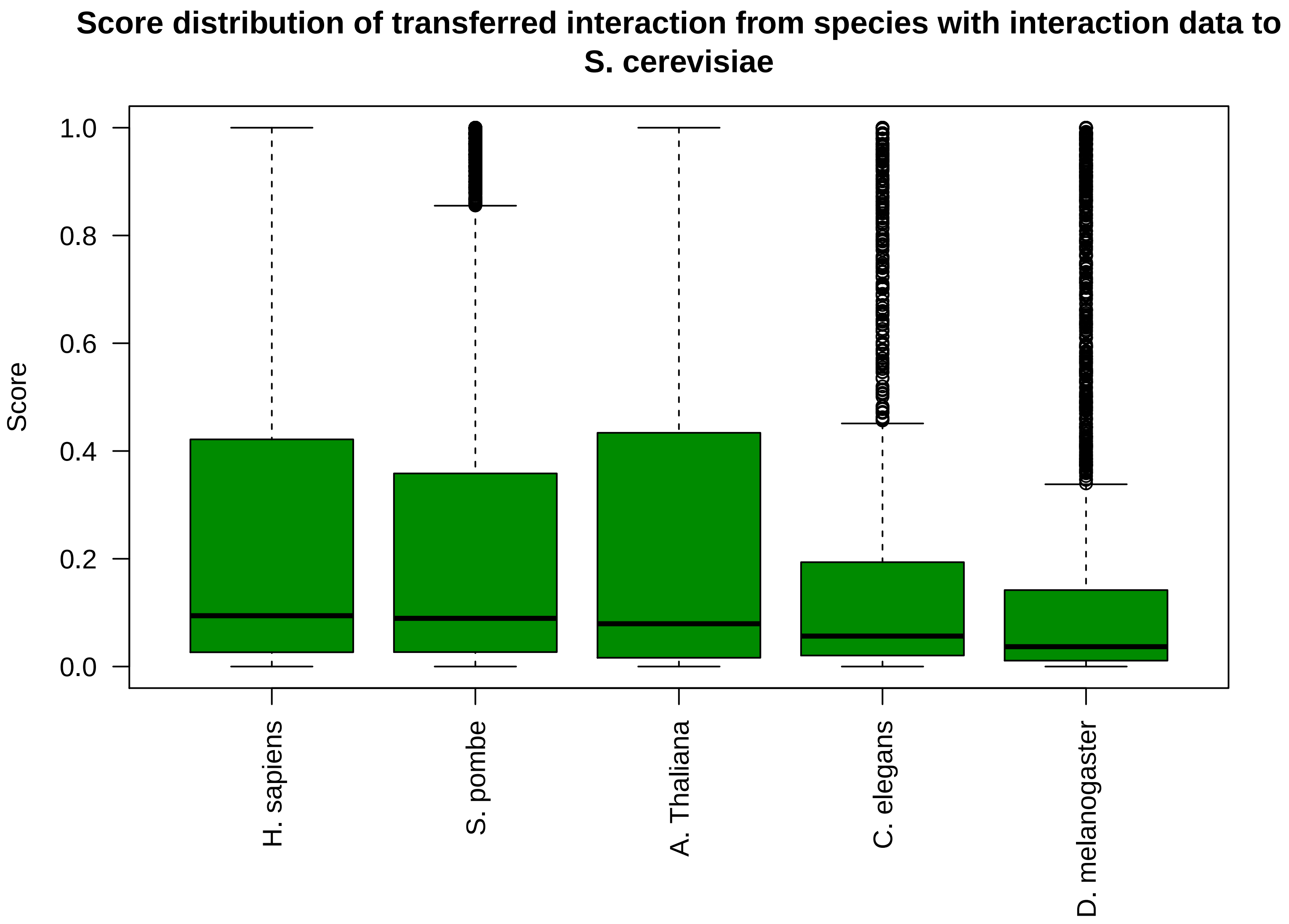
Score distribution of transferred interaction from species with interaction data to S. cerevisiae
Score
1.0
0.8
0.6
0.4
0.2
0.0
H. sapiens
S. pombe
A. Thaliana
C. elegans
D. melanogaster
